# Relationship Between Best Tumor Shrinkage and Progression‐Free Survival and Overall Survival in Patients With Progressive Midgut Neuroendocrine Tumors Treated With [ ^177^Lu]Lu‐DOTA‐TATE: Ad Hoc Analysis of the Phase III NETTER‐1 Trial

**DOI:** 10.1002/cam4.70744

**Published:** 2025-04-24

**Authors:** Marianne Pavel, Martyn E. Caplin, Philippe Ruszniewski, Marianna Hertelendi, Eric P. Krenning, Jonathan R. Strosberg, the NETTER‐1 Study Group

**Affiliations:** ^1^ Department of Medicine 1, Uniklinikum Erlangen and Comprehensive Cancer Center CCC‐EMN Friedrich Alexander University Erlangen‐Nürnberg Erlangen Germany; ^2^ Neuroendocrine Tumour Unit Royal Free Hospital London UK; ^3^ Université Paris Cité and Department of Pancreatology and Digestive Oncology, Beaujon Hospital Clichy France; ^4^ Novartis AG Basel Switzerland; ^5^ Cyclotron Rotterdam B.V., Erasmus Medical Center Rotterdam the Netherlands; ^6^ GI Oncology, H. Lee Moffitt Cancer Center University of South Florida Tampa Florida USA

**Keywords:** [^177^Lu]Lu‐DOTA‐TATE, neuroendocrine tumor, overall survival, progression‐free survival, tumor shrinkage

## Abstract

**Background:**

In many solid tumors, early tumor shrinkage predicts the durability of treatment response. It is unclear whether this is the case for neuroendocrine tumors treated with peptide receptor radionuclide therapy (PRRT).

**Methods:**

Data from the phase III NETTER‐1 study of [^177^Lu]Lu‐DOTA‐TATE (^177^Lu‐DOTATATE) for the treatment of advanced, well‐differentiated, midgut NETs were used to investigate whether objective tumor shrinkage (local review) with ^177^Lu‐DOTATATE is associated with progression‐free survival (PFS) and overall survival (OS) duration.

**Results:**

Overall, 117 patients were treated with ^177^Lu‐DOTATATE (four cycles of 7.4 GBq every 8 weeks). In a landmark analysis, best tumor shrinkage from baseline until data cut‐off (prior to first progression) was not associated with PFS (*n* = 102; hazard ratio: 1.002 [95% confidence interval (CI): 0.99–1.02]; nominal *p *= 0.7808). In further ad hoc analyses, patients on the ^177^Lu‐DOTATATE arm were dichotomized into ≥ 30% tumor shrinkage from baseline (18/117 [15.4%]) and < 30% shrinkage (99/117 [84.6%]). Median (95% CI) PFS was 17.6 (16.5–30.3) months in the ≥ 30% shrinkage group and 25.0 (19.4–31.0) months in the < 30% group. OS was not significantly different for the two tumor shrinkage groups (not estimable [31.0 months–not estimable] and 44.3 [34.9–53.8] months, respectively).

**Conclusions:**

These results suggest the benefit of PRRT and the potential PFS and OS benefit of ^177^Lu‐DOTATATE should not be based on tumor shrinkage (objective response versus stable disease) and that lack of tumor shrinkage should not impact application of the approved four cycles of ^177^Lu‐DOTATATE.

## Graphical abstract

We analyzed data from the phase III NETTER‐1 study of ^177^Lu‑DOTATATE for patients with advanced midgut neuroendocrine tumors to explore whether tumor shrinkage is associated with durable efficacy. Although patients receiving ^177^Lu‑DOTATATE had a progression‐free survival (PFS) benefit, no obvious association between best tumor shrinkage and PFS or overall survival was evident. Our results suggest that disease stabilization is an acceptable outcome of ^177^Lu‑DOTATATE treatment and should not lead to early termination of peptide receptor radionuclide therapy.

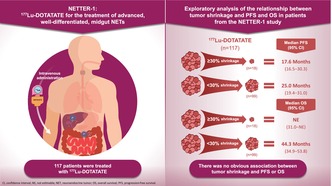

**Abbreviations**
CI: confidence interval
GEP‐NET: gastroenteropancreatic neuroendocrine tumor
HR: hazard ratio
LAR: long‐acting repeatable
NET: neuroendocrine tumor
OS: overall survival
PCHG: percentage change from baseline
PFS: progression‐free survival
PRRT: peptide receptor radionuclide therapy
RECIST: Response Evaluation Criteria in Solid Tumors
SSA: somatostatin analog
SSTR: somatostatin receptor
SWOG: Southwest Oncology Group

## 1 Introduction

Tumor size reduction forms the basis of the Response Evaluation Criteria in Solid Tumors (RECIST) criteria that have been used for decades to evaluate treatment effects on solid tumors [1, 2]. In many tumor types, significant tumor shrinkage is associated with durable response and better outcomes, and early tumor shrinkage has been proposed as a prognostic factor for survival [3, 4, 5, 6, 7, 8]. The relationship between tumor shrinkage and peptide receptor radionuclide therapy (PRRT) outcome in neuroendocrine tumors (NETs) is not so obvious and poses a number of challenges [9, 10, 11]. For example, assessment by morphological change may not be ideal for slow‐growing tumors, and the development of intralesional fibrosis following PRRT can result in tumors appearing to remain the same size or even increase despite improvements in progression‐free survival (PFS) and quality of life [12, 13, 14, 15, 16].

Most NETs express somatostatin receptors (SSTRs), which can be used as a therapeutic target [17, 18, 19]. Lutetium‐177 [^177^Lu]Lu‐DOTA‐TATE (hereafter “^177^Lu‐DOTATATE”), a radiolabeled somatostatin analog, is indicated for the treatment of advanced SSTR‐positive gastroenteropancreatic (GEP) NETs [20, 21, 22]. Approval was based on results from the pivotal NETTER‐1 trial, an open‐label, multicenter, phase III trial of patients with advanced, well‐differentiated, progressive, SSTR‐positive midgut NETs [23, 24]. ^177^Lu‐DOTATATE plus octreotide long‐acting repeatable (LAR) 30 mg significantly prolonged median PFS compared with the control arm of high‐dose octreotide LAR (not reached vs. 8.5 months; hazard ratio [HR] 0.18 [95% confidence interval (CI): 0.11–0.29]; *p* < 0.0001) and demonstrated a significantly greater objective response rate of 18% versus 3% with high‐dose octreotide LAR (*p* < 0.001) as assessed by RECIST v1.1. Overall survival (OS) with ^177^Lu‐DOTATATE plus octreotide LAR 30 mg was 11.7 months longer than that observed with high‐dose octreotide LAR but was not statistically significant.

Studies suggest that, for NETs, PFS and OS benefits of treatment can be observed without the specified 30% tumor size reduction required for a partial response as defined by RECIST [11, 25, 26, 27]. The NETTER‐1 trial represents a unique example of a large randomized clinical trial that offers the opportunity to further explore whether tumor shrinkage on ^177^Lu‐DOTATATE treatment is associated with more durable efficacy and superior PFS and/or OS.

## 2 Materials and Methods

### 2.1 Study Design and Patients

NETTER‐1 was an international, multicenter, open‐label, randomized controlled phase III trial (ClinicalTrials.gov identifier: NCT01578239) to evaluate the efficacy and safety of ^177^Lu‐DOTATATE in patients with advanced, well‐differentiated, SSTR‐positive midgut NETs with disease progression during treatment with octreotide LAR. Full eligibility criteria, study design, and procedures have been described in detail previously [23]. Briefly, 231 patients were randomized 1:1, aged ≥ 18 years with advanced, well‐differentiated inoperable midgut NET Grade 1 or Grade 2 (Ki67 index ≤ 20%). At baseline, patients had confirmed radiological disease progression by RECIST v1.1 while taking octreotide LAR at a standard dose (30 mg/month) and positive uptake on OctreoScan. Patients were enrolled between September 6, 2012, and January 14, 2016. The primary analysis used a data cut‐off date of June 30, 2016, and for this ad hoc analysis we used the final analysis data cut‐off date of January 18, 2021.

### 2.2 Treatment

Patients were randomly assigned to either the ^177^Lu‐DOTATATE group (four cycles of 7.4 GBq every 8 weeks [intravenous infusion] plus best supportive care of intramuscular octreotide LAR 30 mg every 4 weeks) or the control group (treated with high‐dose octreotide LAR 60 mg every 4 weeks). Concomitant amino acid infusion was administered for renal protection. Treatment continued until disease progression, occurrence of intolerable adverse events, or withdrawal of consent. After 72 weeks, patients still in the study were to proceed to the long‐term survival follow‐up phase.

### 2.3 Assessments

Tumor response (change from baseline in the sum of target lesion diameters) was evaluated using computed tomography or magnetic resonance imaging every 12 weeks. During long‐term follow‐up, local radiographic assessments were performed every 6 months.

#### 2.3.1 Landmark Analysis (Tumor Shrinkage and PFS by Time Point)

Best tumor shrinkage was calculated based on the best percentage change from baseline (PCHG) in the sum of target lesion diameters (based on local scan assessment) until each of the following time points and on or before first progression or censoring: 150 days (~21 weeks), 180 days (~26 weeks), 36 weeks, 48 weeks, 72 weeks, and no limit (study cut‐off). Patients whose best response was progressive disease (defined as a change in the sum of target lesion diameters of ≥ 20% and/or appearance of new lesions) were excluded from the analysis. Duration of PFS was determined from the relevant time point to first disease progression or death from any cause, i.e., with the exception of the “no limit” time point (where the duration of PFS was calculated from randomization to first progression or censoring), the “baseline” was reset at the relevant time point. Patients who were not still at risk of progression at this time point were excluded, i.e., patients who discontinued the study, died, progressed, or who were missing post‐baseline measurements prior to the relevant time point. For each treatment arm, the best response in terms of PCHG in the sum of target lesion diameters was used as a covariate in a Cox regression analysis to explain the rate of PFS endpoint events (disease progression or death from any cause). HRs, 95% CIs, and event probability estimates at different time intervals were determined separately for the two treatment arms.

#### 2.3.2 Analysis of PFS and OS by Dichotomized Tumor Shrinkage

In further ad hoc analyses, patients on the ^177^Lu‐DOTATATE arm of the study were categorized into two tumor shrinkage groups based on PCHG: “at least 30% decrease” and “less than 30% decrease”. For these analyses, the intent‐to‐treat principle was used, i.e., patients with a baseline target lesion diameter but no post‐baseline results were grouped in the “< 30% decrease” category. PFS and OS were analyzed by tumor shrinkage groups. This analysis was not performed on the control group due to the small number of patients achieving a tumor shrinkage of at least 30%.

## 3 Results

### 3.1 Patient Population and Baseline Tumor Characteristics

The NETTER‐1 full analysis set (defined as all patients who underwent randomization) included 231 randomized patients, with 117 assigned to the ^177^Lu‐DOTATATE group and 114 to the control group. Patient demographics and disease characteristics were balanced between the treatment groups and have been described previously [23, 24]. The mean age (SD) of study participants was 63 (9) years in the ^177^Lu‐DOTATATE arm and 64 (10) years in the control arm, and 54% and 47% of participants were male, respectively. Overall, 11/117 (9.4%), 35/117 (29.9%), and 71/117 (60.7%) patients in the ^177^Lu‐DOTATATE arm and 13/114 (11.4%), 34/114 (29.8%), and 67/114 (58.8%) patients in the control arm had a Krenning score of 2, 3, or 4, respectively, as defined by the highest tumor uptake on OctreoScan, while under treatment with octreotide LAR (20 to 30 mg every 3 to 4 weeks for a maximum period of 3 years) [24].

### 3.2 Best Percentage Change From Baseline

Patient numbers eligible for analysis at week 72 decreased to 52/117 (44.4%) in the ^177^Lu‐DOTATATE group, and a faster decline was seen in the control group, with 19/114 (16.7%) eligible at the same time point. In the follow‐up phase, 64/117 (54.7%) patients in the ^177^Lu‐DOTATATE arm and 49/114 (43.0%) in the control arm were eligible for assessment at 6 months; the number of patients eligible for assessment dropped to < 10% by 60 months in the ^177^Lu‐DOTATATE arm and 48 months in the control arm.

Figure S1 shows the median best tumor shrinkage at each time point by treatment arm. The median (minimum, maximum) of best PCHG prior to progression at any time point up to week 12 was 0 (−38.5, 19.2) for the ^177^Lu‐DOTATATE group (*n* = 99) and 2.9 (−54.6, 18.0) for the control group (*n* = 80). Up to week 48, the median of best PCHG was −12.3 (−60.0, 11.4) for the ^177^Lu‐DOTATATE group (*n* = 66) and 0 (−46.4, 8.5) for the control group (*n* = 31). Up to week 72, the median of best PCHG was −10.8 (−64.0, 11.4) and − 10.6 (−46.4, 5.0) for the ^177^Lu‐DOTATATE (*n* = 49) and control (*n* = 14) groups, respectively.

**FIGURE 1:**
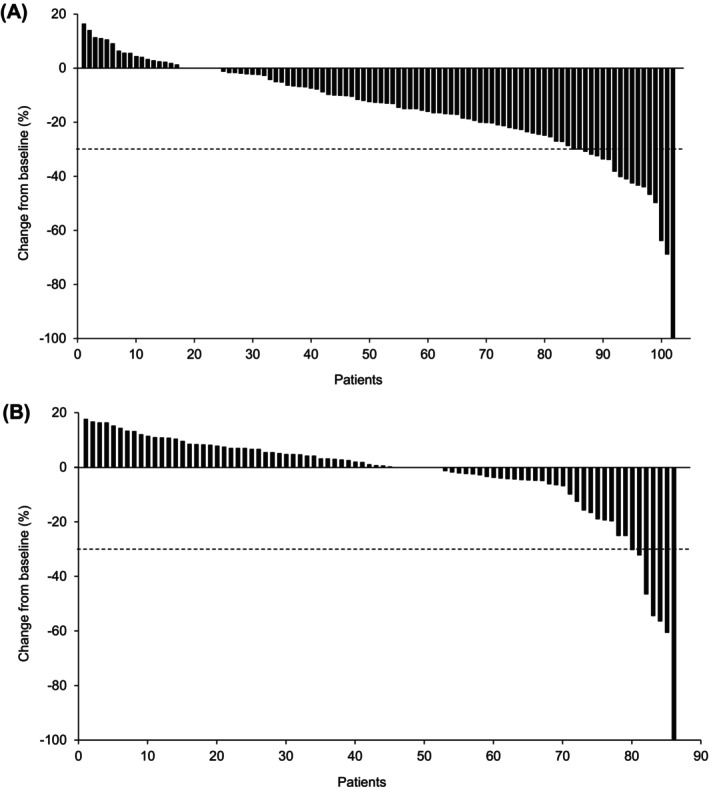
Waterfall plot of best tumor shrinkage (local review) from baseline for (A) ^177^Lu‐DOTATATE group (*n* = 102) and (B) control group (*n* = 86) (full analysis set). Seven patients in the control group had at least a 30% tumor reduction, but only four of these were considered a partial response due to censoring, progression of non‐target lesions, and progression at a later time point.

The best PCHG for each evaluable patient is shown in Figure 1. Most patients (78/102, 76.5%) treated with ^177^Lu‐DOTATATE experienced some form of tumor shrinkage, but only 18 patients showed a change of at least 30%. Fewer patients in the control group showed any tumor shrinkage (34/86, 39.5%), and only seven patients had at least 30% tumor reduction by local review. Results from central analysis, available up to week 72, did not differ significantly from those from local analysis (data not shown).

### 3.3 Relationship Between Best Tumor Shrinkage (Continuous Scale) and PFS

**FIGURE 2:**
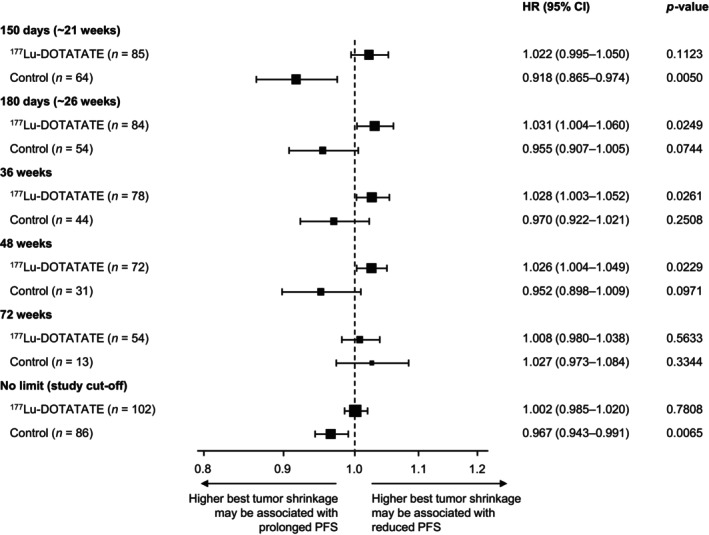
Forest plot showing the impact of a one‐unit increase in best tumor shrinkage on the hazard rate for PFS by time point (full analysis set). Best tumor shrinkage was calculated based on the best percentage change from baseline in the sum of target lesion diameters (based on local scan assessment) until each of the time points and on or before first progression. PFS was determined from the relevant time point to first disease progression or death from any cause. An HR < 1.0 suggests a one‐unit increase in best tumor shrinkage may be associated with a decreased hazard rate for PFS (i.e., prolonged PFS), while an HR > 1.0 suggests a one‐unit increase in best tumor shrinkage may be associated with an increased hazard rate for PFS (i.e., reduced PFS). Control: High‐dose octreotide LAR 60 mg every 4 weeks. CI, confidence interval; HR, hazard ratio; LAR, long‐acting repeatable; PFS, progression‐free survival.

The association between PFS and tumor shrinkage was analyzed by treatment group. In the ^177^Lu‐DOTATATE group, PFS did not appear to be significantly associated with tumor shrinkage (Figure 2). HRs were approximately 1.0 for all time points analyzed suggesting that ^177^Lu‐DOTATATE therapy prolonged PFS even when tumor shrinkage was minimal during treatment cycles. In the control group, tumor shrinkage between baseline and ~21 weeks was associated with improved PFS (*n* = 64; HR 0.918 [95% CI: 0.865–0.974]; *p* = 0.005) (Figure 2). At later time points with lower patient numbers, this association was not as evident, although the relationship remained significant when no time limit (until data cut‐off) was used (*n* = 86; HR 0.967 [95% CI: 0.943–0.991]; *p* = 0.0065).

Tumor shrinkage from baseline for evaluable patients (those who have both baseline and post‐baseline measurements) was plotted versus PFS in Figure S2.

### 3.4 Analysis of PFS and OS by Dichotomized Best Tumor Shrinkage

In the ^177^Lu‐DOTATATE group, 18/117 (15.4%) patients had tumor shrinkage ≥ 30%, and 99/117 (84.6%) patients had tumor shrinkage < 30%. Baseline disease characteristics were similar among the two tumor shrinkage groups (Table S1).

**FIGURE 3:**
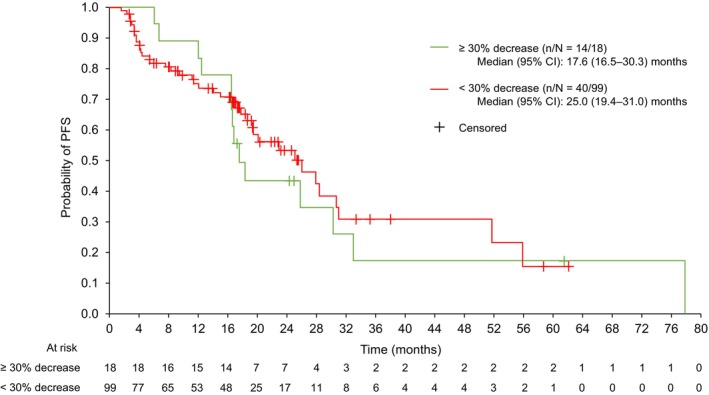
Kaplan–Meier of PFS by tumor shrinkage (local review) up to study cut‐off for ^177^Lu‐DOTATATE group (*n* = 117) (full analysis set). CI, confidence interval; PFS, progression‐free survival.

The median (95% CI) PFS was 17.6 (16.5–30.3) months for the ≥ 30% tumor shrinkage group and 25.0 (19.4–31.0) months for the < 30% decrease group (Figure 3). Overlapping CIs indicated that tumor shrinkage had no significant association with PFS.

**FIGURE 4:**
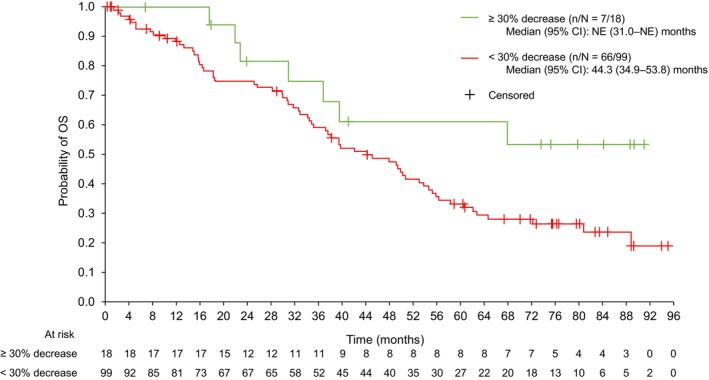
Kaplan–Meier of OS by tumor shrinkage (local review) up to study cut‐off for ^177^Lu‐DOTATATE group (*n* = 117) (full analysis set). CI, confidence interval; NE, not estimable; OS, overall survival.

Similar tumor shrinkage analyses were performed for OS (Figure 4). Median (95% CI) OS was not estimable (31.0 months–not estimable) for the ≥ 30% tumor shrinkage group and 44.3 (34.9–53.8) months for the < 30% decrease group for patients treated with ^177^Lu‐DOTATATE. The CIs overlapped, indicating that tumor shrinkage had no significant association with OS.

Results from central analysis, available up to week 72, showed minor variations without any significant differences (data not shown).

## 4 Discussion

The aim of this study was to use the data from the phase III NETTER‐1 trial to evaluate the relationship between tumor shrinkage and PFS and OS in patients with SSTR‐positive, advanced midgut NETs. PRRT has previously been shown to result in similar OS benefits in patients with gastroenteropancreatic NETs (GEP‐NETs) who had stable disease compared with those achieving a tumor response (complete and partial response) [11, 28]. Conversely, in a larger cohort of patients with bronchial NETs and GEP‐NETs, a clear tendency for improved OS outcomes was observed for patients achieving an objective best tumor response compared with those with stable disease only [9].

The NETTER‐1 trial provided an opportunity to further assess this in a large, well‐defined prospective trial focused on patients with advanced, progressive, well‐differentiated, SSTR‐positive midgut NETs.

In the NETTER‐1 trial, ^177^Lu‐DOTATATE treatment was found to prolong median PFS compared with high‐dose octreotide LAR treatment (not reached vs. 8.5 months; HR 0.18 [95% CI: 0.11–0.29]; *p* < 0.0001) [20, 23]. In the current ad hoc analyses, there was no obvious association between best tumor shrinkage and PFS in the ^177^Lu‐DOTATATE arm. When tumor shrinkage was dichotomized into < 30% decrease and ≥ 30% decrease, no relationship between tumor shrinkage and PFS or OS was observed among patients treated with ^177^Lu‐DOTATATE.

These results are not altogether surprising. We might hypothesize that the lack of correlation between objective response and PFS/OS is because objective response is more likely to occur in higher proliferating tumors (which shrink by a greater percentage but then may progress more quickly) and stable disease is more likely to be the best response in more indolent tumors. However, an additional study would be required to investigate any such association. Objective tumor shrinkage may not occur quickly, or indeed at all, for very slow‐growing NETs [10, 12, 13, 29]. However, we believe it is more likely that the mechanism of action of ^177^Lu‐DOTATATE and the time required to observe best response were responsible for the overall lack of association between PFS and best tumor shrinkage in the ^177^Lu‐DOTATATE group, given that higher best tumor shrinkage (up to data cut‐off) was associated with prolonged PFS in the high‐dose octreotide LAR control group. It remains to be further elucidated why a subset of patients with midgut NETs that received high‐dose octreotide LAR responded with objective tumor shrinkage that corresponded with durable benefit. However, for patients receiving ^177^Lu‐DOTATATE, tumor size reduction on imaging can be modest and best response may take up to a year to be seen [28, 30]. Therefore, we included later time points of up to 18 months in our analysis. Practically, the mechanism of action of ^177^Lu‐DOTATATE may cause heterogeneous tumor effects that can influence imaging measurements and subsequent tumor size assessments. Resulting necrosis and/or fibrosis can look like a stabilization or even an increase in tumor size on a scan [10, 13, 14, 15, 31, 32, 33]. Although it was not investigated in this study, it is possible that tumor necrosis and/or fibrosis may account, at least in part, for the relatively low response rate; however, this would require further investigation.

There are numerous reports in the literature demonstrating PFS benefits with targeted therapies in patients with NETs without objective tumor response, as defined by RECIST criteria. With targeted agents used in the treatment of NETs, including somatostatin analogs (SSAs), everolimus, or tyrosine kinase inhibitors, objective response rates are low (< 10%) [26, 27, 34]. Studies with everolimus for advanced NETs found a PFS benefit with only a low proportion of patients showing an objective tumor response (2%–5%) [27, 34]. Similar results have been observed with the SSA lanreotide when used to treat patients with metastatic pancreatic/intestinal NETs [25]. Consistent with our results, an analysis of 268 patients with GEP‐NETs and thoracic NETs treated with ^177^Lu‐DOTATATE found that patients with stable disease had similar PFS and OS as patients with tumor regression based on four response scoring systems (RECIST [≥ 30% decrease cut‐off for tumor regression], Southwest Oncology Group [SWOG; ≥ 50% cut‐off] solid tumor response criteria, modified RECIST [≥ 13% cut‐off], and modified SWOG [≥ 25% cut‐off] criteria) [11]. Approximately half of these patients (54%) were diagnosed with midgut NETs, akin to those enrolled in NETTER‐1.

Our study does have certain limitations. This analysis was not part of the originally planned endpoints of NETTER‐1, and therefore caution should be applied when interpreting the results more generally. The study only deals with a midgut NET population, and higher responses may be observed in patients with other NET primaries. Studies have suggested that the primary location of NETs can influence the amount of tumor shrinkage observed with PRRT, with pancreatic NETs (foregut) showing more shrinkage/objective response than those located in the midgut [9, 35]. Additionally, the RECIST definition of objective tumor response (≥ 30% decrease in the sum of diameters of target lesions) is quite rigorous, and given the potential for tumor necrosis/fibrosis following PRRT, even minor tumor shrinkage may have relevance [1, 10, 13, 14, 15, 31, 32, 33]. Although consistent results were found on central analysis within a shorter observational period, our analyses are based on local review at multiple centers, which may inherently introduce some variation despite the application of a detailed protocol. Finally, the trial population size may have been insufficient to demonstrate a correlation between objective response and PFS or OS.

These data have implications for clinical practice. With regard to midgut NETs, and based on the available analysis, achieving objective tumor shrinkage should not guide the use of PRRT with respect to the number of treatment cycles. We confirm that response evaluation with RECIST criteria has limitations for assessment of the efficacy of PRRT with ^177^Lu‐DOTATATE for patients with NETs. A treatment benefit exists even if the best response after a full course of treatment is stable disease.

In conclusion, the potential PFS/OS benefits of ^177^Lu‐DOTATATE cannot be clearly predicted based on the occurrence of tumor shrinkage. A 30% cut‐off per RECIST criteria and best tumor shrinkage were not associated with PFS for patients with midgut NETs treated with ^177^Lu‐DOTATATE. Despite this, ^177^Lu‐DOTATATE appeared to prolong PFS in this population even when tumor shrinkage was minimal during treatment cycles; therefore, lack of or minimal tumor shrinkage should not impact application of the approved four cycles of ^177^Lu‐DOTATATE.

## Author Contributions

**Marianne Pavel:** conceptualization (equal), formal analysis (equal), writing – review and editing (equal). **Martyn E. Caplin:** conceptualization (equal), formal analysis (equal), writing – review and editing (equal). **Philippe Ruszniewski:** conceptualization (equal), formal analysis (equal), writing – review and editing (equal). **Marianna Hertelendi:** formal analysis (equal), writing – review and editing (equal). **Eric P. Krenning:** conceptualization (equal), formal analysis (equal), writing – review and editing (equal). **Jonathan R. Strosberg:** conceptualization (equal), formal analysis (equal), writing – review and editing (equal).

Data were analyzed by the sponsor (Novartis Pharma AG) and results provided to all authors for further interpretation. The work reported in the paper has been performed by the authors, unless clearly specified in the text.

All authors contributed to the interpretation of the results and critical revision of the manuscript.

## Ethics Statement

The NETTER‐1 study is registered at ClinicalTrials.gov, NCT01578239. The study protocol was approved by independent ethics review boards at each site. All patients provided written informed consent.

## Conflicts of Interest

Marianne Pavel: Received honoraria for presentations and/or acted as a consultant for Advanced Accelerator Applications, a Novartis Company; Ipsen; Novartis Pharma AG; Eli Lilly; Riemser; Boehringer Ingelheim; MSD; Hutchmed; Serb; Sanofi; Esteve; Tairix; and ITM Radiopharma, and serves as an adviser for SMC of Crinetics and SSC of Novartis. Martyn E. Caplin: Received honoraria for presentations and/or acted as a consultant for Advanced Accelerator Applications, a Novartis Company; Crinetics; Ipsen; and Pfizer. Philippe Ruszniewski: Scientific adviser to Advanced Accelerator Applications, a Novartis Company; Ipsen; and ITM Radiopharma. Marianna Hertelendi: Full‐time employee of Novartis Basel and holds shares in Novartis. Eric P. Krenning: Retired (Erasmus MC, Rotterdam, Netherlands)—previous shareholder of Advanced Accelerator Applications, a Novartis Company/BioSynthema. Jonathan R. Strosberg: Consulted for Novartis; institutional trial support from ITM and Radiomedix.

## Supporting information


Data S1.

## Data Availability

Novartis is committed to sharing with qualified external researchers access to patient‐level data and supporting clinical documents from eligible studies. These requests are reviewed and approved by an independent review panel on the basis of scientific merit. All data provided are anonymized to respect the privacy of patients who have participated in the trial in line with applicable laws and regulations. This trial data availability is according to the criteria and process described on www.clinicalstudydatarequest.com.
