# Supplementary material for: Relationship Between Best Tumor Shrinkage and Progression‐Free Survival and Overall Survival in Patients With Progressive Midgut Neuroendocrine Tumors Treated With [ 177Lu]Lu‐DOTA‐TATE: Ad Hoc Analysis of the Phase III NETTER‐1 Trial
Source: Cancer Med. 2025 Apr 24;14(9):e70744. doi: 10.1002/cam4.70744 (PMC12020026; doi:10.1002/cam4.70744)
Supplement: Supplementary file 1 — Data S1. [file CAM4-14-e70744-s001.docx]

**SUPPLEMENTARY MATERIAL**

Relationship between best tumor shrinkage and progression-free survival and overall survival in patients with progressive midgut neuroendocrine tumors treated with [177Lu]Lu‑DOTA-TATE: *Ad hoc* analysis of the phase III NETTER-1 trial

Marianne Pavel*, Martyn E. Caplin, Philippe Ruszniewski, Marianna Hertelendi, Eric

P. Krenning, Jonathan R. Strosberg, on behalf of the NETTER-1 study group

*Corresponding author: [Marianne.Pavel@uk-erlangen.de](mailto:Marianne.Pavel@uk-erlangen.de)

**SUPPLEMENTARY FIGURE S1** Best tumor shrinkage (local review) by timepoint and treatment arm (FAS).


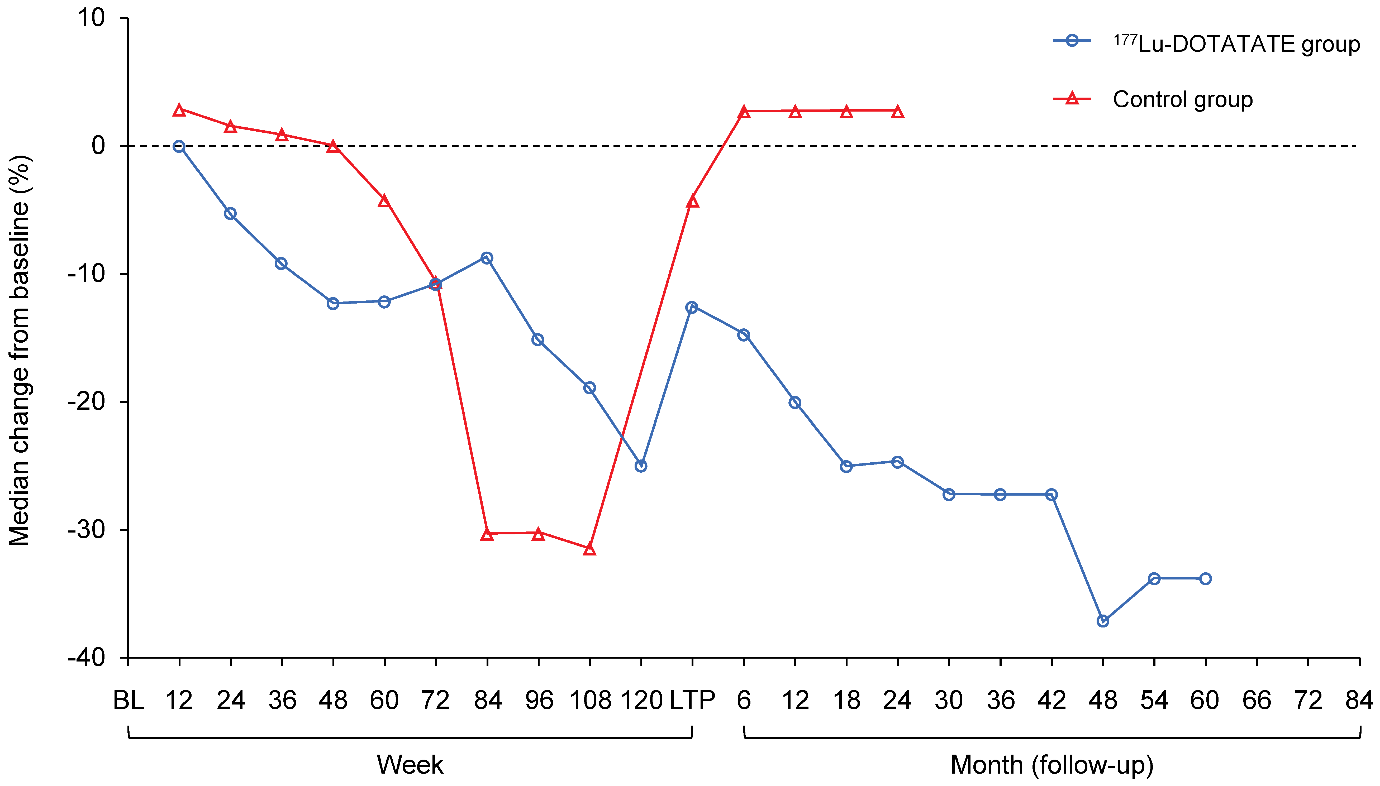


Best tumor shrinkage was calculated based on best percentage change from baseline in the sum of target lesion diameters (based on local scan assessment) until each of the timepoints and on or before first progression.

At later timepoints, data were based on a sample size of ≤20 patients per treatment group. This applied to the control group at week 72, both treatment groups at weeks 84–120, the control group at the LTP, and both treatment groups for all timepoints during the follow-up period. At the LTP, the analysis included 35 patients from the ^177^Lu-DOTATATE group and 15 patients from the control group.

Abbreviations: BL, baseline; FAS, full analysis set; LTP, last treatment period.

**SUPPLEMENTARY FIGURE S2** Scatter plot of best tumor shrinkage (local review) versus PFS for (A) 177Lu‑DOTATATE group (n = 102) and (B) control group (n = 86) (FAS).


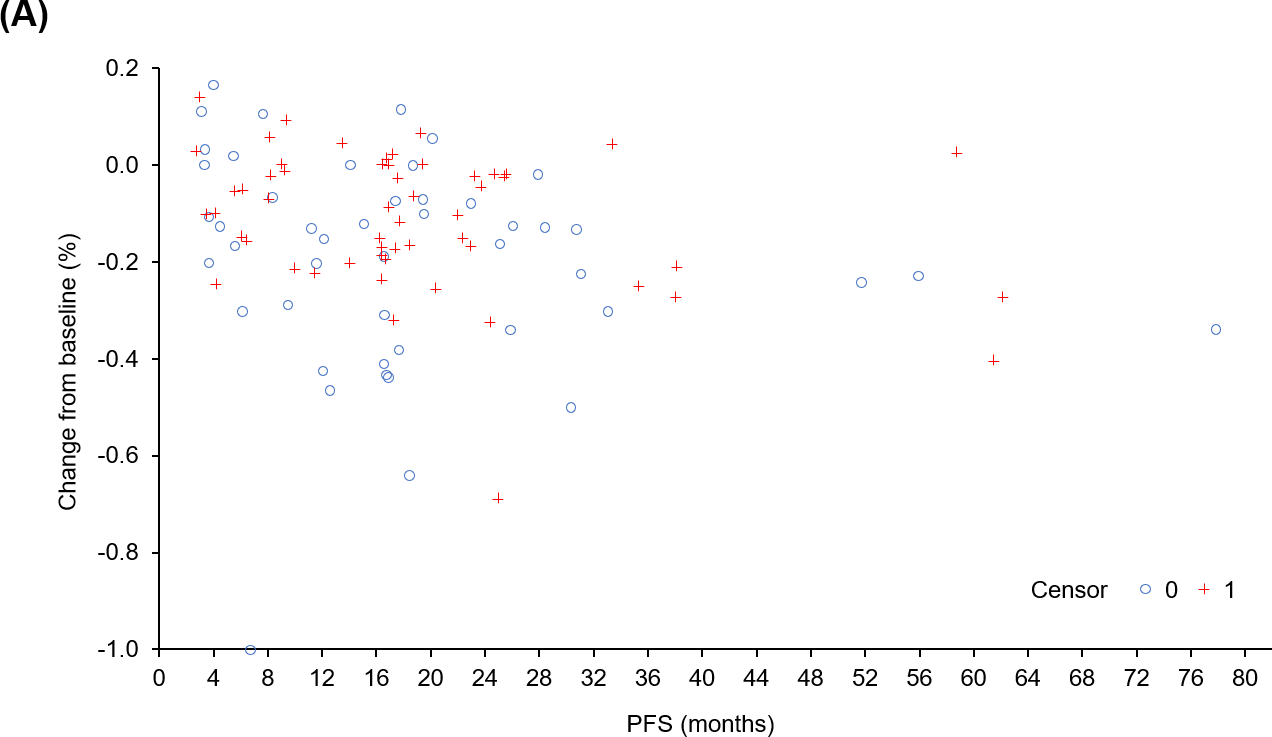


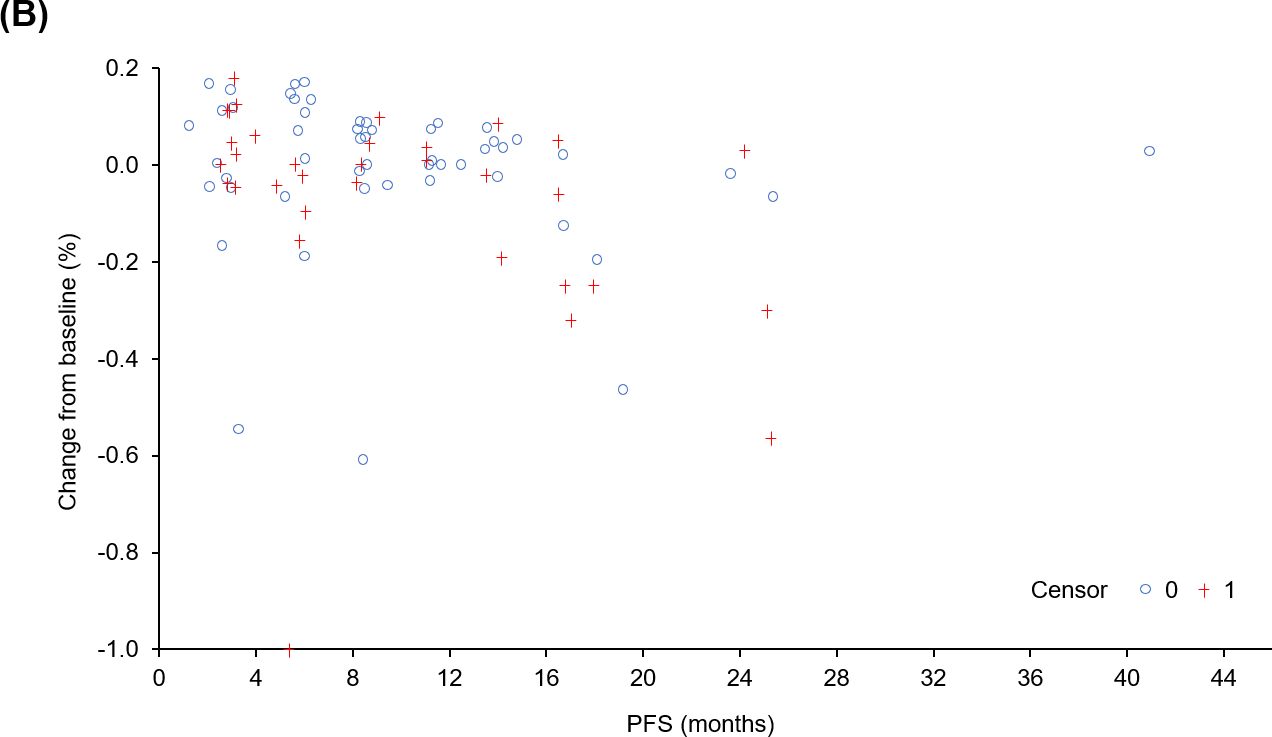


Abbreviations: FAS, full analysis set; PFS, progression-free survival.

**SUPPLEMENTARY TABLE S1** Baseline disease characteristics by tumor shrinkage (local review) for ^177^Lu‑DOTATATE group (n = 117) (FAS).

|  | ^177^Lu‑DOTATATE n = 117 | |
| --- | --- | --- |
| Baseline characteristic | ≥30% decrease n = 18 | <30% decrease n = 99 |
| OctreoScan highest tumor uptake grade, n (%) |  |  |
| Grade 2 | 2 (11.1) | 9 (9.1) |
| Grade 3 | 7 (38.9) | 28 (28.3) |
| Grade 4 | 9 (50.0) | 62 (62.6) |
| Time on constant dose octreotide, n (%) |  |  |
| ≤6 months | 6 (33.3) | 25 (25.3) |
| >6 months | 12 (66.7) | 74 (74.7) |
| Karnofsky index, n (%) |  |  |
| ≤80 | 6 (33.3) | 26 (26.3) |
| 90 | 6 (33.3) | 45 (45.5) |
| 100 | 6 (33.3) | 27 (27.3) |
| Missing | 0 | 1 (1.0) |
| Previous therapies, n (%) | 15 (83.3) | 82 (82.8) |
| Chemotherapy | 2 (11.1) | 8 (8.1) |
| Radiotherapy | 0 | 4 (4.0) |
| Surgery | 15 (83.3) | 79 (79.8) |
| Tumor burden, n (%) |  |  |
| Limited | 15 (83.3) | 85 (85.9) |
| Moderate | 3 (16.7) | 10 (10.1) |
| Extensive | 0 | 4 (4.0) |
| Ki67 index, n (%) |  |  |
| ≤2% | 12 (66.7) | 65 (65.7) |
| 3 to 20% | 6 (33.3) | 34 (34.3) |
| CgA level, n (%) |  |  |
| ≤2xULN | 6 (33.3) | 12 (12.1) |
| >2xULN | 9 (50.0) | 52 (52.5) |
| Missing | 3 (16.7) | 35 (35.4) |
| 5-HIAA level, n (%) |  |  |
| ≤2xULN | 6 (33.3) | 28 (28.3) |
| >2xULN | 7 (38.9) | 48 (48.5) |
| Missing | 5 (27.8) | 23 (23.2) |
| Alkaline phosphatase level, n (%) |  |  |
| ≤ULN | 11 (61.1) | 61 (61.6) |
| >ULN | 5 (27.8) | 36 (36.4) |
| Missing | 2 (11.1) | 2 (2.0) |

Abbreviations: 5-HIAA, 5-hydroxyindole acetic acid; CgA, chromogranin A; FAS, full analysis set; ULN, upper limit of normal.
